# Swimming training and *Plantago psyllium* ameliorate cognitive impairment and glucose tolerance in streptozotocin–nicotinamide-induced type 2 diabetic rats

**DOI:** 10.1186/s12576-021-00823-z

**Published:** 2021-11-27

**Authors:** Hesam Parsa, Zahra Moradi-Khaligh, Sara Rajabi, Kamal Ranjbar, Alireza Komaki

**Affiliations:** 1 Department of Exercise Physiology, Faculty of Sport Sciences, Bu-Ali Sina University, Hamedan, Iran; 2 Department of Physical Education and Sport Science, Bandar Abbas Branch, Islamic Azad University, Bandar Abbas, Iran; 3 Neurophysiology Research Center, Hamadan University of Medical Sciences, Hamadan, Iran

**Keywords:** Swimming training, *Plantago psyllium*, Type 2 diabetes, Learning and memory, Metabolic disorders, Rat

## Abstract

Brain malfunction is common in diabetic patients. On the other hand, a growing body of research points to the beneficial effect of medicinal plants and exercise training on insulin sensitivity and brain function. Therefore, the aim of the present study was to investigate the effect of co-administration of swimming training and *Plantago psyllium* (mixed with standard pelleted food at a weight ratio of 5%) on learning and memory impairment and glucose tolerance in type 2 diabetic rats. For this purpose, 10 healthy and 40 rats with type 2 diabetes were randomly allocated to five groups: healthy sedentary control group (Con), sedentary diabetic group (D), diabetic group subjected to swimming training (D + Tr), diabetic group receiving *P. psyllium* (D + Ps), and diabetic group subjected to swimming training and receiving *P. psyllium* (D + Ps + Tr). Diabetes was induced by a single intraperitoneal injection of nicotinamide (120 mg/kg) and streptozotocin (65 mg/kg) separately with 15 min intervals. Experimental groups were treated with swimming training and *P. psyllium* independently and simultaneously for 12 weeks. Lipid profile and food intake were measured and also, glucose tolerance was evaluated by glucose area under the curve (AUCg) using an oral glucose tolerance test. Passive avoidance learning (PAL) and memory were evaluated by shuttle box test and cognitive memory was assessed by novel object recognition (NOR) and elevated plus-maze (EPM) tests. Diabetic rats exhibited a significant increase in food intake, lipid profile, and AUCg compared to healthy rats. Step-through latency in the PAL acquisition trial (STL-a) and retention test (STL-r) were significantly lower in diabetic rats than in the control group. In the diabetic group without treatment, time spent in the dark compartment increased compared to the control group in the shuttle box test. Discrimination index and distance traveled reduced in diabetic rats. On the other hand, swimming training and *P. psyllium* alleviated food intake, lipid profile, and glucose tolerance in diabetic rats. Also, the STL-a, STL-r, discrimination index, and distance travelled in the D + Ps + Tr group were significantly more than the diabetic group. Results showed that 12 weeks of swimming training and receiving *P. psyllium* improved memory deficit in streptozotocin–nicotinamide-induced type 2 diabetic rats possibly through hypolipidemic and hypoglycemic effects. These results suggest that the administration of swimming training and *P. psyllium* simultaneously might be an effective intervention for the treatment of diabetes-induced behavioral deficits.

## Introduction

Diabetes mellitus is the most common group of heterogeneous metabolic and inflammatory disorders that its prevalence has risen dramatically in the twenty-first century in developed and developing countries. According to the World Health Organization (WHO) [1], the total number of patients with diabetes is projected to rise from 171 million in 2000 (2.8%) to 366 million in 2030 (4.4%), and also the prevalence of diabetes in adults is predicted to rise to 10.4% (642 million) in 2040 [2, 3].

Dyslipidemia and dysfunctional glucose are the common complications of patients with type 2 diabetes that is characterized by elevated levels of cholesterol, low-density lipoprotein (LDL), and triglyceride (TG), and decreased levels of high-density lipoprotein (HDL) that result from insufficient and defective insulin secretion, insulin resistance, or both [4]. Dyslipidemia and abnormalities in carbohydrate and lipid metabolism and also insulin resistance lead to cognitive impairments and are risk factors for the development of Alzheimer's disease (AD) and dementia in diabetic patients. Metabolic disorders affect the morphology and plasticity of the hippocampus and result in cognitive deficits [5, 6].

The origin of cognitive deficits in diabetes is not yet clear [7]. It has been reported that memory deficits are related to the functional and structural deficit in the central neuron system [8]. In this regard, reduced cerebral blood flow and amplitude of low-frequency fluctuations in brain infarcts and the amygdala increased volume of the ventricular and white matter, impaired network integrity, abnormal microstructure and atrophy of the whole brain, particularly the grey matter and hippocampus, as well as alterations in glutamate neurotransmission were observed in diabetic patients with cognitive dysfunction [9, 10]. Also, hyperglycemia induces the overproduction of pro-inflammatory and inflammatory cytokines (neuro-inflammation) and toxic accumulation of amyloid-beta (Aβ) [6], and eventually, these changes cause cell death [11].

Exercise training regimens are the main non-pharmacological strategy to reduce various difficulties related to mood disorders and metabolic diseases. Exercise training has been suggested to prohibit cognitive deficits caused by different chronic disorders affecting neurological function [12, 13]. In this regard, previous studies have confirmed that exercise training, especially aerobic training raises cell proliferation in the dentate gyrus [14] and hippocampal plasticity [15], up-regulates brain-derived neurotrophic factor (BDNF) and neuron growth factor (NGF) and down-regulates the genes associated with oxidative stress [12, 16]. Most studies have used running training [17–19] to assess the effect of exercise training on diabetes-induced neurodegenerative diseases, and the protective or offensive role of swimming training in memory performance is not yet clear.

On the other hand, recently, the focus has shifted to the use of herbs for the treatment of diabetic complications due to their lower side effects [20]. *Plantago major* L. is one of these medical plants with anti-hyperglycemic, lipid-lowering, and hypolipidemic effects. It is a member of the Plantaginaceae family with two medicinal species of Isabgol (*Plantago ovata*) and psyllium (*Plantago psyllium*) [21]. Psyllium is native to the Mediterranean area and is high in both fiber and mucilage (10%–15%) [22]. A meta-analysis published in 2015 reported that consuming psyllium before meals reduced fasting blood glucose (FBS) concentration (−37.0 mg/dL) and glycated hemoglobin (HbA1c) [−0.97% (−10.6 mmol/mol)] in subjects under treatment for type 2 diabetes [23]. Also, psyllium fiber decreased LDL and TG in type 2 diabetic patients [24]. The beneficial effect of dietary *P. psyllium* on memory deficit in diabetic patients has not yet been demonstrated.

Therefore, for the first time, the present study was designed to evaluate the possible ameliorative effects of swimming training and *P. psyllium* on the cognitive malfunction, insulin sensitivity, and lipid profile in type 2 diabetic rats.

## Methods

### Animals

Fifty male albino Wistar rats (200–250 g, 6–8 weeks old) were prepared from the animal house of the Hamedan University of Medical Science. They were adapted to the environment for 1 week prior to the initiation of the experiments. All rats were caged in conventional conditions (12:12 h light:dark cycle, temperature: 22 ± 2 ℃, and humidity: 50 ± 5%) with ad libitum access to purified water and standard chow.

### Experimental design

**Fig. 1:**
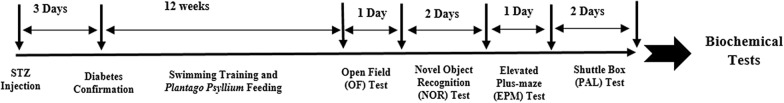
The experimental timeline. The type 2 diabetes model was induced by a single intraperitoneal injection of nicotinamide (120 mg/kg) and streptozotocin (60 mg/kg), and confirmed by a fasting glucose level of ≥ 250 mg/dL three days later. Swimming training began one day after confirmation of diabetes and the rats underwent 12 weeks of progressive swimming training. During swimming training, *Plantago psyllium*-treated groups received *P. psyllium* mixed with standard pelleted food at a weight ratio of 5% for 12 weeks. For the measurement of locomotion, an open field test was employed, for the assessment of cognitive memory, the novel object recognition (NOR) and elevated plus-maze (EPM) tests were used, and for the measurement of aversive (acquisition and retention) learning and memory after the training programs, the shuttle box test was used

Ten healthy and 40 diabetic rats were randomly allocated to five groups: healthy sedentary control group (Con, *n* = 10), sedentary diabetic group (D, *N* = 10), diabetic group subjected to swimming training (D + Tr, *n* = 10), diabetic group receiving *P. psyllium* (D + Ps, *n* = 10), and diabetic group subjected to swimming training and receiving *P. psyllium* (D + Ps + Tr, *n* = 10) (Fig. 1). Animal Care Committee for Laboratory Animal Research of the Hamedan University of medical science reviewed and approved all animal experiments in this study.

### Diabetic induction and blood glucose monitoring

After the adaptation period, all experimental rats were deprived of food for 12 h before each injection for diabetes induction. A single dose of streptozotocin (STZ; 65 mg/kg) (Sigma-Aldrich, Saint Louis, MO) dissolved in 0.1 mol citrate buffer (pH 4.5) was intraperitoneally injected into the rats. Then, before STZ injection, nicotinamide (120 mg/kg, soluble in normal saline) was injected intraperitoneally to the rats after the fasting period. STZ was injected after 15 min of nicotinamide administration. Healthy rats were injected with the same dose of buffer solution. After 72 h, blood was taken from the tail area, and fasting blood sugar levels were ascertained by a glucometer (Arkray Glucocard 01 mini). Animals with fasting blood sugar levels above 250 mg/dL were considered diabetic rats.

### Swimming training protocol

The exercise training protocol was carried out as mentioned in other papers [25, 26] with slight modifications. Animals in the training groups were subjected to progressive freestyle swimming in temperature-controlled (32 ± 2 °C) swimming pools for 12 weeks. The swimming exercise was performed in plastic barrels (45 cm in diameter) filled with water to a depth of 50 cm. For alleviating stress and adaptation to swimming, animals were adapted to water prior to initiating the experiment for 1 week (5 sessions per week, each session 10 min) with no addition of a weight to the tail. Rats were swimming in the pool alone during the light cycle. The initial exercise sessions during the first week were performed for 15 min and 5 days per week. In the second to the seventh week, in the overload phase, the length of the swimming exercise training gradually was increased to 30 min per day, and from the eighth to the twelfth week, the exercise duration was kept constant (40 min/day, 5 days/week) with 2 days of rest. During swimming, a weight corresponding to 2% of the body weight was attached to the rats’ tails.

### *Plantago psyllium* preparation and food intake evaluation

*Plantago psyllium* was collected from the local market of Hamedan and approved by the Department of Pharmacy of the Hamedan University of Medical Science. In Iran, most research centers provide normal animal food from Pars Animal Feed Company, which contains (100 g of the food) carbohydrates (57 g), protein (17.5 g), fat (2 g), fiber (6.6 g), vitamins and mineral s(4.9 g), and energy (316 kcal). Five grams of *P. psyllium* was mixed with 1 l of water to obtain a homogeneous mixture, and then 95 g of normal food was added to the mixture, followed by mixing with a mixer to obtain a homogeneous dough. *P. psyllium* was mixed with standard pelleted food at a weight ratio of 5%. The dough was then poured into a meat grinder for making rat food pellets and the rat pellets were obtained. The pellets were placed in a covered environment and after drying, and the rats were fed with them. To ensure the consumption of *P. psyllium* by rats, the weight of the remained rat's food was recorded. For food intake measurement, food weight was subtracted from its amount in the previous day at a specific time each day. Also, the rats’ weights were also measured on a specific day each week.

### Open field test

In order to assess the general locomotor activity and exploratory behavior of rats, an open field test was applied [27]. Briefly, the apparatus is a 100 × 100 × 40 cm hypethral box with the bottom divided into four identical squares on the floor of the arena. Each rat was positioned in the center of the cage and the distance travelled as locomotor activity and exploratory behavior [28] were observed over 5 min using the EthoVision video tracking system (Noldus, Leesburg, VA, USA).

### Novel objects recognition (NOR) test

Novel object recognition (NOR) test was used for assessing non-spatial and the hippocampal-dependent recognition memory of treated and non-treated rats [29–31]. This test is used to measure cognitive memory in rodents and is based on the integrity of the hippocampus. The test was done within 3 days: habituation day, training day, and testing day as previously described [32, 33] with some minor modifications. During training, conditions were provided for the rat to explore two identical objects. On the test day, one of the training objects was replaced by another object that was different in appearance (shape and color). Exploration time of each object (time spent sniffing or touching the object but not leaning against or sitting or standing on the object) was recorded. The discrimination index was also calculated as follows: DI = (TNO–TFO)/(TNO + TFO) × 100), where TNO is the exploration time of the novel object and TFO is the exploration time of the familiar object [33, 34]. It should be noted that all sessions were video recorded and analyzed blindly.

### Elevated plus-maze (EPM) test

The anxiolytic activity was evaluated by the elevated plus-maze (EPM) test. As previously described [35], the EPM apparatus consists of two opposing closed arms (10 × 50 cm) and two opposing open arms (50 × 10 × 50 cm) connected by a central square (10 × 10 cm), and the maze is elevated 80 cm from the floor. Each rat was placed in the center of the apparatus facing one of the closed arms and allowed to search inside the apparatus for 600 s. Video recording of each rat was later analyzed for time spent in closed arms. After each trial, the apparatus was cleaned with 10% ethyl alcohol. It should be noted that all behavioral assessments were performed during the light cycle.

### Passive avoidance learning (PAL) test: shuttle box

Passive avoidance memory was evaluated by the shuttle box test according to our previous papers [12, 13, 36–38]. The device had lighted and dark compartments similar in dimension (20 × 20 × 30 cm), with a grid stainless-steel rod floor connected to a shock generator and a guillotine door separating two compartments. At first, in order to acclimatize in the acquisition trial, the rat was placed in a lighted section and then, the guillotine door was opened. Thirty seconds after the rat entered the dark section, it was returned to its home cage. After 30 min, this test was repeated. When the animal had the whole body in the dark section, the entrance latency to the dark section in the acquisition trial (step-through latency, STL-a) was measured. The guillotine door between two sections was closed and then, an electrical shock was applied (0.8 mA) to the rat for 2 s. Thirty seconds after receiving the electrical shock, the rat was returned to its home cage. The test was carried out again after 2 min. Each time the rat re-entered the dark section, it received an electric shock. When the rat stayed in the light section for 120 s, the test was finalized and the number of trials was recorded [12, 13, 39].

The retention test was executed 24 h following the PAL acquisition trial. In this phase, the rat was placed in the light section and the guillotine door was opened for 5 s, and then, the step-through latency in retention test (STL-r) and the time spent in the dark section (TDC) were measured for 600 s [13, 40].

### The oral glucose tolerance test (OGTT)

One of the most common methods for assessing glucose homeostasis and insulin sensitivity in rodents is the OGTT. Briefly, 24 h after the shuttle box test, the rats were administered with glucose solution (2 g/kg b.w.) by oral gavage following overnight fasting. At 0, 30, 60, 90, and 120 min after the glucose load, plasma glucose was measured. The glucose area under curve (AUCg) during the OGTT was calculated [41].

### Measurement of biochemical parameters

One day after OGTT, blood was collected from the portal vein and centrifuged at 3000 rpm for 10 min at 4 ℃. Serum levels of TG, cholesterol, LDL, HDL, and very low-density lipoproteins (VLDL) were measured by an enzymatic photometric test using Pars Azmoon test kits (Pars Azmoon Co., Iran). Also, the atherogenic index (AI = log (TG/HDL)) was measured. Serum blood glucose concentration was measured by the glucose oxidase method (Pars Azmoon kits, Iran) based on the manufacturer’s instructions. The experimental timeline is shown in Fig. 1.

### Statistical analyses

The data were analyzed using the SPSS version 20.0 (IBM SPSS Statistics). Shapiro–Wilk test was used to determine the normal distribution of data. The statistical difference between the groups was estimated using one-way ANOVA and Tukey’s post hoc test. A 5 × 4 mixed-plot factorial repeated measures ANOVA was performed to analyze for differences in 0, 30, 60 and 120 min after the glucose load, plasma glucose between the groups. The data were expressed as mean ± standard error of the mean. A *p*-value of 0.05 was considered statistically significant.

## Results

### Open field test

**Fig. 2:**
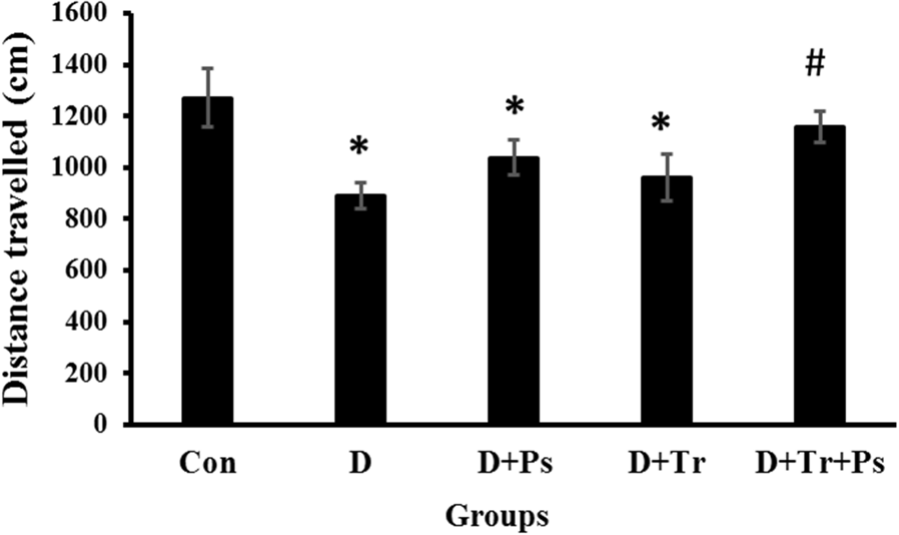
Comparison of the experimental groups regarding the distance travelled as locomotor activity and exploratory behavior in the open field test. *P. psyllium* and swimming training synergistically increased locomotor activity and exploratory behavior compared to the control diabetic group. Values are presented as mean ± SEM. # Significant difference compared to the diabetic rats (*p* ≤ 0.05) and * the significant difference compared to the control group (*p* ≤ 0.05)

The distance traveled was different between experimental groups (*F* = 2.96, *p* = 0.03). The results showed that there was a significant decrease in distance travelled in the diabetic group compared to the control group (*p* = 0.02). However, distance travelled was significantly lower in the D + Ps and D + Tr groups compared to the control group (*p* = 0.01 and *p* = 0.01, respectively, Fig. 2). Furthermore, the diabetic rats subjected to swimming and receiving the herb had higher activity scores compared to the diabetic group (*p* = 0.01). There was no significant difference between the control and D + Tr + Ps groups in this regard (*p* = 0.89).

### Novel object recognition test

**Fig. 3:**
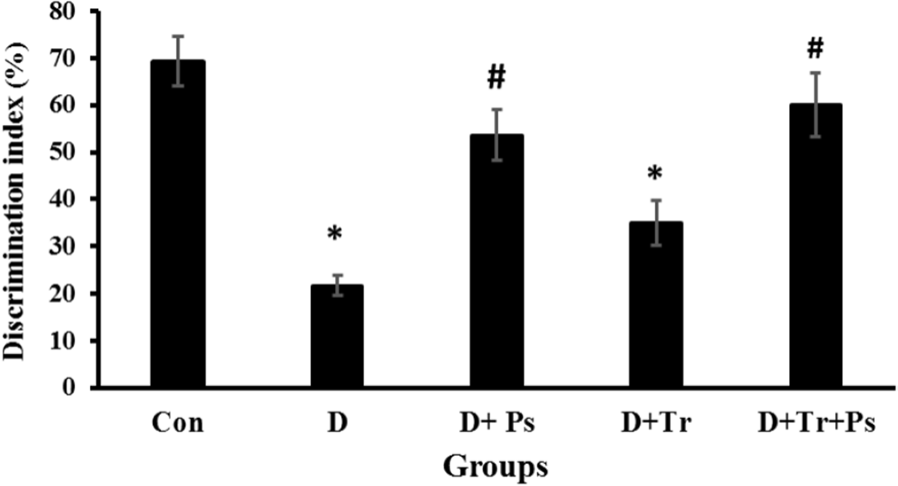
Comparison of the groups regarding the discrimination index in the novel object recognition test. *Plantago psyllium* intake for 12 weeks with and without swimming training promoted the discrimination index, but swimming training alone had no effect on the discrimination index in this test. Values are presented as mean ± SEM. # Significant difference compared to the diabetic group (*p* ≤ 0.05) and * significant difference compared to the control group (*p* ≤ 0.05)

Statistical analyses showed that DI was different between experimental groups (*F* = 45.3, *p* = 0.0001). As shown in Fig. 3, untreated diabetic rats showed a significant decrease in DI (*p* = 0.0001) compared to the control group. Treatment with *P. psyllium* significantly increased DI similar to the rats treated with *P. psyllium* that were subjected to swimming training compared to the diabetic control rats (*p* = 0.0001 and *p* = 0.0001, respectively). DI in the D + Tr + Ps group was more than that of the D + Ps group, but this difference was not statistically significant (*p* = 0.08). Furthermore, swimming exercise insignificantly promoted DI in diabetic rats (*p* = 0.06).

### Elevated plus-maze test

**Fig. 4:**
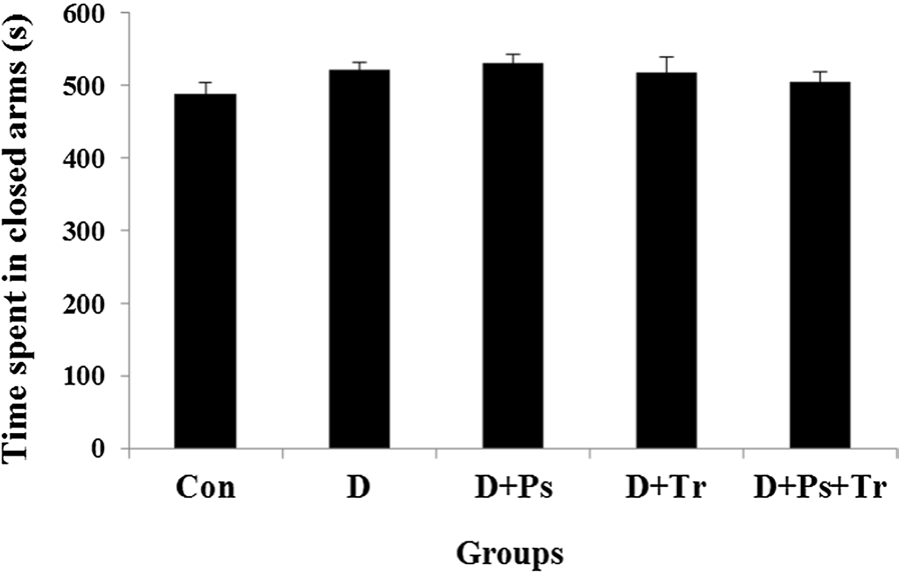
Anxiolytic activity using the elevated plus-maze (EPM) test evaluated by the time spent in closed arms in diabetic rats subjected to swimming training and receiving *Plantago psyllium*. There was no significant difference between the experimental groups. Values are presented as mean ± SEM

As shown in Fig. 4, statistical analyses showed that time spent in closed arms was not significantly different between the experimental groups (*F* = 0.54, *p* = 0.9).

### Passive avoidance learning test

**Fig. 5:**
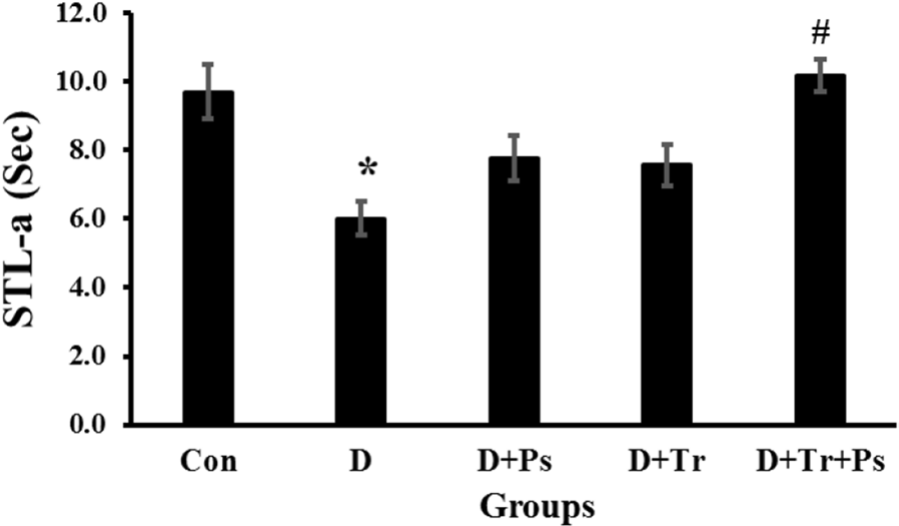
Comparison of the groups regarding step-through latency in the acquisition trial (STL-a) in the shuttle box test. Diabetic rats had a lower STL-a compared to the healthy rats. Swimming training and consumption of *Plantago psyllium* elevated STL-a compared to the diabetic rats. Values are presented as mean ± SEM. # Significant difference compared to the diabetic rats (*p* ≤ 0.05) and * significant difference compared to the control group (*p* ≤ 0.05)

As demonstrated in Fig. 5, statistical analyses showed that STL-a was different between groups (*F* = 6.01, *p* = 0.0001). STL-a was significantly lower in diabetic rats than in the control group (0.003). Swimming training and treatment with *P. psyllium* caused a significant increase in the STL-a compared to the diabetic rats (*p* = 0.003). Furthermore, there were no significant differences between the D + Ps and D + Tr groups compared to the control (respectively, *p* = 0.18, *p* = 0.16) and diabetic (respectively, *p* = 0.34, *p* = 0.52) rats in the acquisition trial.

**Fig. 6:**
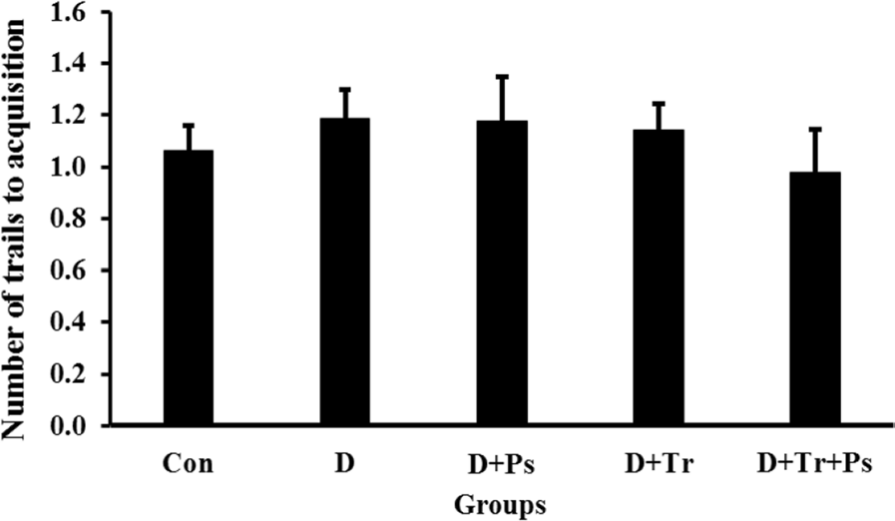
The number of trials to acquisition between the experimental groups. There was no significant difference between the experimental groups; which means that swimming training and *P. psyllium* had no effect on the number of trials to acquisition in the PAL test. Values are presented as mean ± SEM

As shown in Fig. 6, the number of trials to acquisition was not significantly different between the experimental groups (F = 0.35, p = 0.83). Although the number of trials to acquisition was more in the diabetic rats (*p* = 0.96) and slightly decreased in the D + Tr + Ps group, these differences were not significant (*p* = 0.87).

**Fig. 7:**
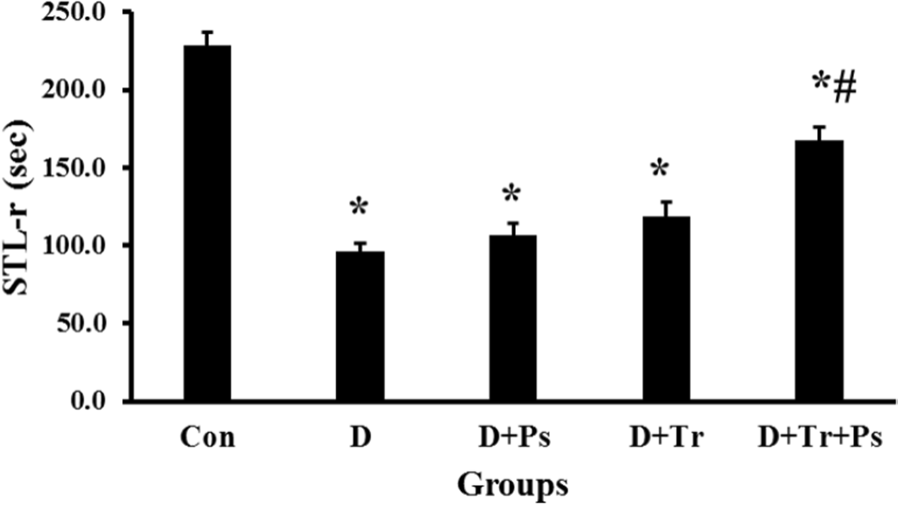
Step-through latency in the retention phase (STL-r) of the passive avoidance learning test. Swimming training and *Plantago psyllium* synergistically ameliorated STL-r, but the treatments alone had no effect on STL-r in diabetic rats. Values are presented as mean ± SEM. # Significant difference compared to the diabetic rats (*p* ≤ 0.05) and * significant difference compared to the control group (*p* ≤ 0.05)

STZ-induced diabetes affected memory retention as shown in Fig. 7. Statistical analyses showed differences in STL-r between the experimental groups (*F* = 61.17, *p* = 0.0001). The results indicated that STL-r of diabetic animals significantly reduced compared to the control group (*p* = 0.0001). Regarding the effect of swimming training and *P. psyllium* on the learning ability of the diabetic animals, STL-r was found with a significant increase, which was significant (0.0001) in the group subjected to swimming training and receiving *P. psyllium* simultaneously.

**Fig. 8:**
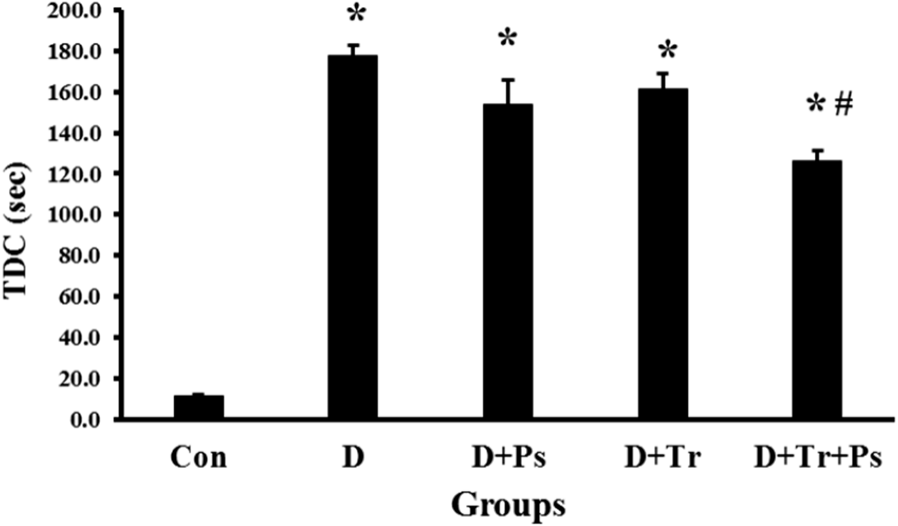
Comparison of the groups regarding the time spent in the dark compartment (TDC). Diabetic rats stayed in the dark compartment longer than healthy rats and swimming training and *Plantago psyllium* synergistically mitigated TDC compared to the other diabetic groups. Values are presented as mean ± SEM. # significant difference compared to the diabetic rats (*p* ≤ 0.05) and * significant difference compared to the control group (*p* ≤ 0.05)

Comparison of TDC in the shuttle box test between diabetic and control rats revealed significant differences between the groups (*F* = 117.4, *p* = 0.0001, Fig. 8). In the diabetic group without treatment, TDC significantly increased compared to the control group (*P* = 0.0001). Swimming training and *P. psyllium* treatment independently had no effect on TDC in diabetic rats (*p* = 0.44 and *p* = 0.1, respectively)*.* Treatment of diabetic rats subjected to swimming training with *P. psyllium* simultaneously reduced the TDC (*p* = 0.0001).

### Food intake and AUCg in type 2 diabetic rats

**Fig. 9:**
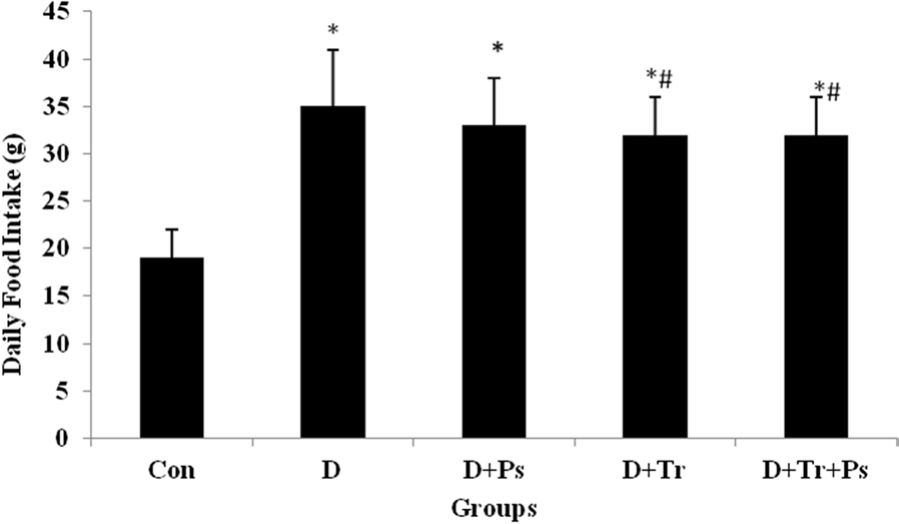
Daily food intake in the control and diabetic rats. Analysis of variance revealed a significant difference between the experimental groups. The food intake in diabetic rats was almost twice compared to the healthy control rats. Values are presented as mean + SEM. # Significant difference compared to the diabetic rats (*p* ≤ 0.05) and * significant difference compared to the control group (*p* ≤ 0.05)

Statistical analyses showed that daily food intake was significantly different between the groups (*F* = 78.8, *p* = 0.0001). Diabetic rats showed significantly (*p* = 0.0001) higher intake of food compared to the control group. The food intake in diabetic rats was almost twice compared to the healthy control rats. In this regard, food intake in the training groups was more than healthy control rats (*p* = 0.0001). It was noteworthy that food intake in the training groups with and without *P. psyllium* supplementation significantly decreased compared to the control diabetic rats (*p* = 0.03 and *p* = 0.01, respectively). Daily food intake between the D + Ps, D + Tr, and D + Ps + Tr was not different (Fig. 9).

**Fig. 10:**
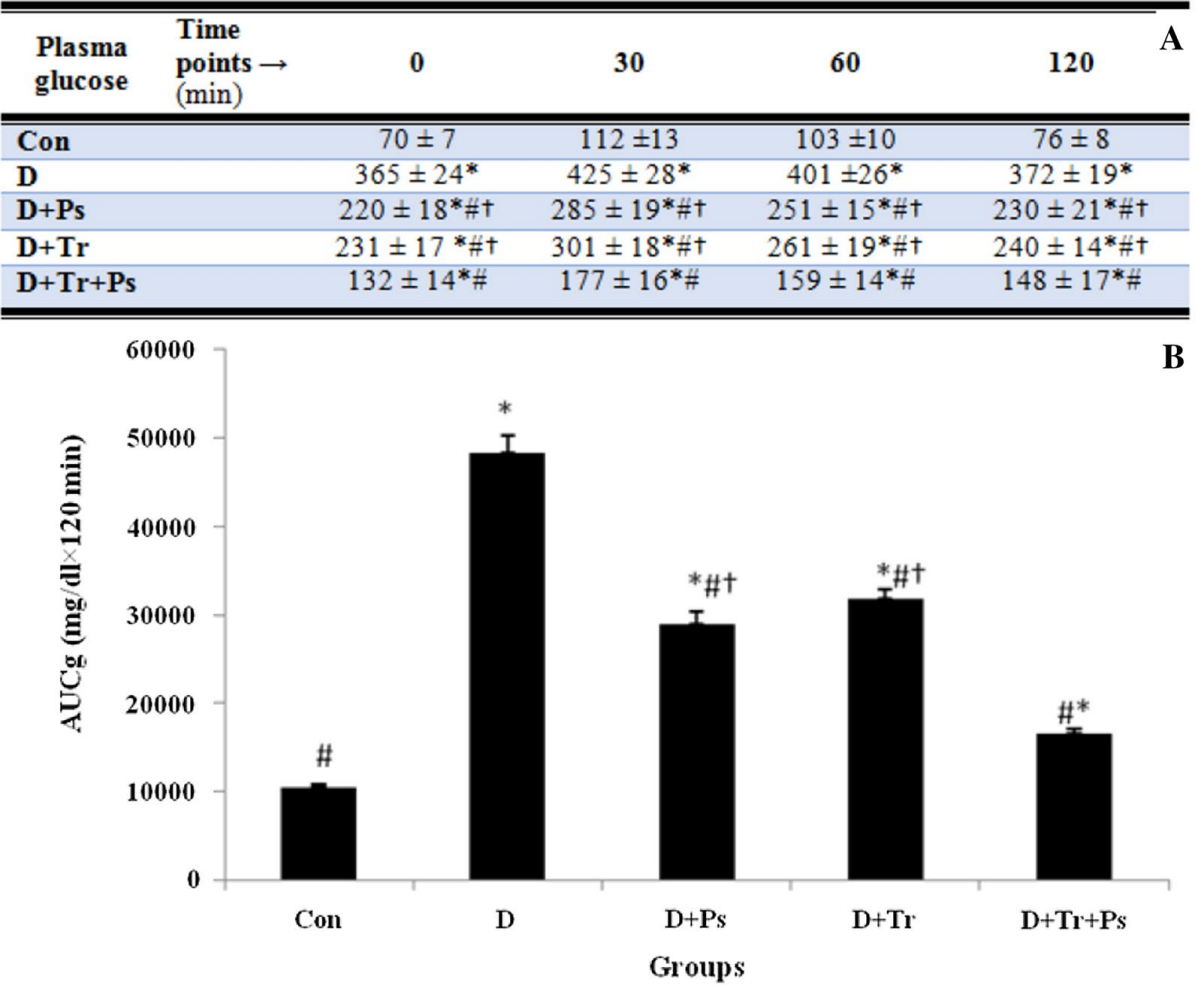
**A** Plasma glucose levels in 0, 30, 60 and 120 min after glucose load in experimental groups and **B** glucose area under the curve in oral glucose tolerance test (OGTT). Analyses showed insulin resistance in diabetic rats was 5 times higher than in healthy rats. Exercise training and P. psyllium independently and simultaneously significantly decreased AUCg in diabetic rats. Values are expressed as mean + SEM. # Significant difference compared to the diabetic rats (*p* ≤ 0.05), * significant difference compared to the control group (*p* ≤ 0.05), and † significant difference compared to the diabetic group subjected to swimming training and receiving *P. psyllium* (D + Ps + Tr group) (*p* ≤ 0.05)

As shown in Fig. 10, AUCg was different between the groups (*F* = 1267.7, *p* = 0.0001). Type 2 diabetic rats had the largest AUCg, and diabetes caused a significant increase in AUCg compared to the healthy rats (*p* = 0.0001). Importantly, consumption of *P. psyllium* by diabetic rats caused a significant reduction (*p* = 0.0001) in the AUCg compared to the diabetic group, and this effect was almost as large as that of diabetic rats that performed only swimming training. Swimming training also significantly reduced (*p* = 0.0001) the AUCg compared to the control diabetic group. Swimming training and *P. psyllium* showed the greatest significant reduction (*p* = 0.0001) in the AUCg so that the amount of AUCg was almost equal to the healthy control rats. AUCg in response to treatments decreased, but it was still more than (*p* = 0.0001) healthy rats. In general, the results showed that between the study groups, the lipid profile in the D + Ps + Tr group was the closest to the lipid profile in healthy rats.

### Serum biochemical parameters

Diabetic rats exhibited a significant (*p* ≤ 0.05) increase in TG, cholesterol, LDL, VLDL, and AI compared to the normal rats. On the other hand, HDL and weight in diabetic rats were less than healthy rats. Swimming training with and without *P. psyllium* consumption ameliorated biochemical parameters in diabetic rats. Swimming training and *P. psyllium* consumption for 12 weeks simultaneously increased HDL (*p* = 0.001) and weight and decreased LDL (*p* = 0.0001), VLDL (*p* = 0.0001), TG (*p* = 0.0001), cholesterol (*p* = 0.0001), and AI in diabetic rats. The changes in the lipid profile and AI of healthy and diabetic rats in different experimental groups are illustrated in Table 1.

**Table 1:** Biochemical parameters in different experimental groups

Variables	Con		D	D + Ps	D + Tr	D + Ps + Tr
Weight (g)	420 ± 14		348 ± 23^b^	401 ± 34^a,b^	352 ± 25^b^	358 ± 15^b^
Fasting glucose (mg/dl)	105.2 ± 9.7		290 ± 18.7^b^	222 ± 12.6^a,b,c^	189.3 ± 13.3^a,b^	180.4 ± 19.2^a,b^
TG (mg/dl)	101.2 ± 13.7		206.8 ± 29.3^b^	185.2 ± 16.3^a,b,c^	136.3 ± 16.3^a,b^	134.4 ± 18.2^a,b^
Cholesterol (mg/dl)	154.2 ± 11.6		241.8 ± 29.3^b^	198.6 ± 12.3^a,b,c^	177.3 ± 12.4^a,b,c^	163.3 ± 13.7^a^
HDL (mg/dl)	37.5 ± 5.8		22.4 ± 4.3^b^	23.3 ± 4.6^b,c^	27.3 ± 3.3^b^	30.6 ± 4.1^a,b^
LDL (mg/dl)	33.6 ± 3.6		53.2 ± 4.1^b^	49.3 ± 3.7^b,c^	45.6 ± 4.2^a,b,c^	37.9 ± 5.6^a^
VLDL (mg/dl)	30.1 ± 4.3		48.4 ± 3.1^b^	45.2 ± 2.9^b^	40.3 ± 5.2^a,b^	33.8 ± 5.6^a^
Atherogenic index	0.43 ± 0.08		0.96 ± 0.08^b^	90 ± 0.05^b^	70 ± 0.03^a,b^	65 ± 0.07^a,b^

Values are presented as mean ± SEM. ^a^Significant difference compared to the diabetic rats (*p* ≤ 0.05), ^b^significant difference compared to the control group (*p* ≤ 0.05), and ^c^significant difference compared to the diabetic group subjected to swimming training and receiving *P. psyllium* (D + Ps + Tr group) (*p* ≤ 0.05)

Fasting glucose (*F* = 43.73, *p* = 0.0001) was significantly different between the experimental groups. As shown in Table 1, fasting glucose significantly increased in diabetic rats compared to the healthy rats (*p* = 0.0001). Swimming training and *P. psyllium* consumption independently and simultaneously ameliorated fasting serum glucose in diabetic rats (0.0001).

## Discussion

### Cognitive decline in diabetes

The results showed an increase in daily food intake, serum glucose levels, TG, cholesterol, LDL, and VLDL in streptozotocin–nicotinamide-induced type 2 diabetic rats compared to the healthy rats. In contrast, HDL and insulin sensitivity in diabetic rats were less than healthy rats. Elevated levels of TG and cholesterol in diabetes are related to hippocampal lipid metabolism [42]. It has been reported that diabetes reduces brain abilities. The association between lipid profile elevation and brain function dysfunction has not yet been elucidated, but recently, it has been shown that an increase in lipid profile can partially reduce the integrity of the hippocampus, the primary brain structure for encoding and recalling memories. Hippocampus viscoelasticity is negatively associated with serum TG levels and the TG/HDL-C ratio [43]. In this regard, the results of this study showed that diabetes decreases the general locomotor activity and passive avoidance, and cognitive memory, but anxiolytic activity, despite an insignificant increase, was not affected by diabetes induction. These findings are similar to previous studies, in which diabetes-induced behavioral deficits in rodents have been reported [44, 45]. Also, an overview of prospective observational studies showed that diabetes caused a 1.2- to 1.7-fold increase in the odds of cognitive decline [46]. Patients with diabetes are more susceptible to AD compared to non-diabetic patients [47]. Glucose deregulation and decreased action of insulin are central to the development and progression of dementia in diabetic patients. An elevation in blood glucose levels is associated with cognitive impairment and brain grey matter volume depression [48]. The molecular mechanism of diabetic encephalopathy (pathological brain damages) in diabetic patients is complex and poorly understood. Several mechanisms have been proposed to play a critical role in diabetic encephalopathy and cognitive deficits in STZ-induced diabetic rats. Insulin resistance leads to the formation of neurofibrillary tangles and the deposition of extracellular Aβ plaques. Both pathways facilitated neurodegeneration in diabetic and AD patients. On the other hand, chronic hyperglycemia promotes inflammation and oxidative stress in the hippocampus [49]. Chronic inflammation and oxidative stress can result in neurogenesis reduction, disruption of neuronal functioning, loss of synapses, a decline in neurotransmitter release, and increased neuronal death in the dentate gyrus of the hippocampus and the subventricular zone through damage to the cell membranes, DNA, and protein structure [46]. The decreased number and function of neurons in the hippocampus causes memory loss [50]. Different pathways are involved in the development of dementia in diabetic patients. Malfunction of NMDA and AMPA glutamate receptors, which are involved in cognitive processes, occurs in diabetic patients [51]. Recently, Aleksandra (2020) in a review paper, introduced C-reactive protein (CRP), microRNAs (miRNAs), paraoxonase 1 (PON-1), glycogen synthase kinase 3β (GSK-3β), phosphoinositide 3-kinases (PI3K), amylin, dopamine, gamma-glutamyl transferase (GGT), various growth factors (NGF, TGF, and BDNF), and homocysteine, as the main actors in neurodegeneration in AD, mild cognitive impairment, and dementia [52]. Also, the consequences of elevated lipid levels, such as inflammation, can cause memory deficits in diabetic rats.

### Effect of exercise training on cognitive deficit

Strategies used for the treatment of diabetes can prevent or delay cognitive impairment. Physical activity is one of the most effective strategies. It has become known that exercise training decreased inflammation and stress oxidative, which are the main causes of memory deficit in diabetic patients [53, 54]. The result of this paper showed that 12 weeks of swimming training insignificantly promoted passive avoidance memory (↑26% STL-a, ↑23% STL-r, and ↓9% TDC), locomotor activity, and exploratory behavior (↑7% distance traveled), and non-spatial cognitive memory (↑ 7% DI) compared to the sedentary diabetic rats. Also, exercise training significantly decreased LDL, VLDL, TG, and cholesterol and promoted HDL and insulin sensitivity in streptozotocin–nicotinamide-induced type 2 diabetic rats. Training-induced lipid levels and insulin sensitivity amelioration may improve memory impairment in diabetic rats. In agreement with our study, Mehta et al. [54] showed that exposure to running wheel for 6 weeks reversed diabetes-associated cognitive decline by reduced levels of neuroinflammatory markers (IL-1β, TNF-α, and MCP-1) and hippocampus neuronal density. They concluded that aerobic exercise could partially reverse memory deficit by reducing oxidative stress and inflammation in the brain of animals with type 2 diabetes [54]. Also, our laboratory reported that resistance training for 10 weeks ameliorated cognitive deficit in type 1 diabetic rats. Type, intensity, and duration of training program may be effective interfering factors to determine the success rate of training to reverse memory deficit. In this regard, Zarrinkalam et al. demonstrated that different types of exercise (endurance, resistance, and concurrent) exert similar effects on spatial memory, but they have distinct effects on aversive memory in morphine-dependent rats [13]. Therefore, the type of exercise can be one of the reasons for the discrepancy between the results of this study and other studies.

Different mechanisms have been proposed for the ameliorative effect of exercise training to reverse hyperglycemia-associated neurological complications, such as the management of energy metabolism and synaptic plasticity [55]. A significant increase in hippocampal acetylcholine levels (the primary neurotransmitter responsible for learning and memory) was observed in response to aerobic training [54]. New evidence indicates that hippocampal neurogenesis and neuronal density elevation, blood–brain barrier (BBB) permeability reduction, and gene expression modulation (an elevation in the expression of genes that belong to the synaptic modulation and signal transduction categories, such as BDNF, Orexin-1, and claudin-5) are likely involved in the promotion of brain abilities in response to exercise training in type 2 diabetic patients [55–58].

### Effect of *P. psyllium* on cognitive deficit

The results of this paper showed that long-term *P. psyllium* consumption promoted passive avoidance memory (↑29.6% STL-a, ↑11% STL-r, and ↓13% TDC), locomotor activity, exploratory behavior (↑16.6% distance traveled), and non-spatial cognitive memory (**↑**145% DI) compared to the sedentary diabetic rats. Also, *P. psyllium* decreased TG, LDL, VLDL, and cholesterol levels and increased HDL levels and glucose homeostasis. This is the first study that evaluated the protective effect of *P. psyllium* on behavioral deficit under hyperglycemic condition; however, the molecular mechanism of this protective effect is not clear and needs more studies in the future. The main focus of diabetes management is glycemic control and some herbs, such as *P. psyllium,* are very effective in this regard. *P. psyllium* has been used for the treatment of inflammatory diseases and its anti-hyperglycemic and lipid-lowering effects have been reported in animal studies. Moran et al. and Ziai et al. demonstrated that treatment with *P. psyllium* for 6–8 weeks through decreased intestine glucose uptake reduced plasma lipid and glucose levels in patients with type 2 diabetes [59, 60]. Nevertheless, the exact ingredients in the plant responsible for the protection have not yet been identified and more studies are needed.

### Protective effect of swimming training and *P. psyllium*

The main finding of this study was that co-administration of swimming training and *P. psyllium* reversed memory impairment in streptozotocin–nicotinamide-induced type 2 diabetic rats. Swimming training and *P. psyllium* increased passive avoidance memory (↑69.5% STL-a, ↑74% STL-r, and ↓29% TDC), locomotor activity, exploratory behavior (↑30% distance traveled), and non-spatial cognitive memory (↑172% DI) compared to the sedentary diabetic rats. The important point is that co-administration of swimming training and *P. psyllium* for 12 weeks completely reversed locomotor activity and non-spatial cognitive memory in streptozotocin–nicotinamide-induced type 2 diabetic rats. The values of STL-a, DI, and distance traveled, which were impaired in response to metabolic disease, were not significantly different between the D + Ps + Tr and healthy rats. Swimming training and *P. psyllium* simultaneously ameliorated lipid profile and insulin sensitivity. The concomitant use of the extract and swimming training was more effective than the extract or swimming training alone.

Hypolipidemic and hypoglycemic effects of swimming training and *P. psyllium* are probably due to the activation of PPAR-Y, triggering glucokinase activity in the liver, and inducing glucose transporter-4 expression. Co-administration of swimming training and *P. psyllium* is probably more effective on inflammation and oxidative stress suppression in diabetic rats. As a result, the simultaneous use of these two factors possibly causes more neurogenic effects in the brain of diabetic rats. Lipids inhibit the intracellular signaling pathway of insulin to open glucose channels. It is possible that exercise training and *P. psyllium* simultaneously promoted insulin sensitivity and memory deficit in diabetic rats via decreased lipid levels, consequences such as inflammation, oxidative stress, and viscoelasticity of the hippocampus, and these changes promote brain function.

## Conclusion

According to the obtained results, we concluded that long-term swimming training and receiving *P. psyllium* simultaneously improved memory deficit and insulin sensitivity in streptozotocin–nicotinamide-induced type 2 diabetic rats through the amelioration of lipid profile. These results suggest that swimming training and *P. psyllium* simultaneously might be an effective intervention for the treatment of diabetes-induced behavioral deficit.

## Data Availability

All data and material are available.

## References

[1] Wild S, Roglic G, Green A, Sicree R, King H (2004). Global prevalence of diabetes: estimates for the year 2000 and projections for 2030. Diabetes Care.

[2] Ogurtsova K, da Rocha FJ, Huang Y, Linnenkamp U, Guariguata L, Cho NH, Cavan D, Shaw J, Makaroff L (2017). IDF Diabetes Atlas: global estimates for the prevalence of diabetes for 2015 and 2040. Diabetes Res Clin Pract.

[3] Rababa'h AM, Mardini AN, Alzoubi KH, Ababneh MA, Athamneh RY (2019). The effect of cilostazol on hippocampal memory and oxidative stress biomarkers in rat model of diabetes mellitus. Brain Res.

[4] Pirmoghani A, Salehi I, Moradkhani S, Karimi SA, Salehi S (2019). Effect of Crataegus extract supplementation on diabetes induced memory deficits and serum biochemical parameters in male rats. IBRO Rep.

[5] Wang BN, Wu CB, Chen ZM, Zheng PP, Liu YQ, Xiong J, Xu JY, Li PF, Al Mamun A, Ye LB, Zheng ZL (2021) DL-3-n-butylphthalide ameliorates diabetes-associated cognitive decline by enhancing PI3K/Akt signaling and suppressing oxidative stress. Acta Pharmacologica Sinica. 1–14 10.1038/s41401-020-00583-3 PMC8027654 33462377

[6] McNay EC, Recknagel AK (2011). Reprint of: ‘Brain insulin signaling: a key component of cognitive processes and a potential basis for cognitive impairment in type 2 diabetes’. Neurobiol Learn Mem.

[7] Ghasemi M, Zendehbad B, Zabihi H, Hosseini M, Hadjzadeh MAR, Hayatdavoudi P (2016). Beneficial effect of leptin on spatial learning and memory in streptozotocin-induced diabetic rats. Balkan Med J.

[8] Omidi G, Karimi SA, Rezvani-Kamran A, Monsef A, Shahidi S, Komaki A (2019). Effect of coenzyme Q10 supplementation on diabetes induced memory deficits in rats. Metab Brain Dis.

[9] Zhou J, Zhang Z, Zhou H, Qian G (2020). Diabetic cognitive dysfunction: from bench to clinic. Curr Med Chem.

[10] Nardin P, Zanotto C, Hansen F, Batassini C, Gasparin MS, Sesterheim P, Gonçalves C-A (2016). Peripheral levels of AGEs and astrocyte alterations in the hippocampus of STZ-diabetic rats. Neurochem Res.

[11] Volpe CMO, Villar-Delfino PH, Dos Anjos PMF, Nogueira-Machado JA (2018). Cellular death, reactive oxygen species (ROS) and diabetic complications. Cell Death Dis.

[12] Zarrinkalam E, Ranjbar K, Salehi I, Kheiripour N, Komaki A (2018). Resistance training and hawthorn extract ameliorate cognitive deficits in streptozotocin-induced diabetic rats. Biomed Pharmacother.

[13] Zarrinkalam E, Heidarianpour A, Salehi I, Ranjbar K, Komaki A (2016). Effects of endurance, resistance, and concurrent exercise on learning and memory after morphine withdrawal in rats. Life Sci.

[14] Kim H-B, Jang M-H, Shin M-C, Lim B-V, Kim Y-P, Kim K-J, Kim E-H, Kim C-J (2003). Treadmill exercise increases cell proliferation in dentate gyrus of rats with streptozotocin-induced diabetes. J Diabetes Complications.

[15] Gomes da Silva S, Unsain N, Mascó DH, Toscano-Silva M, de Amorim HA, Silva Araújo BH, Simoes PSR, da Graça N-M, Mortara RA, Scorza FA (2012). Early exercise promotes positive hippocampal plasticity and improves spatial memory in the adult life of rats. Hippocampus.

[16] Stranahan AM, Lee K, Becker KG, Zhang Y, Maudsley S, Martin B, Cutler RG, Mattson MP (2010). Hippocampal gene expression patterns underlying the enhancement of memory by running in aged mice. Neurobiol Aging.

[17] Li J, Liu Y, Liu B, Li F, Hu J, Wang Q, Li M, Lou S (2019). Mechanisms of aerobic exercise upregulating the expression of hippocampal synaptic plasticity-associated proteins in diabetic rats. Neural Plast.

[18] Heidarianpour A, Mohammadi F, Keshvari M, Mirazi N (2021). Ameliorative effects of endurance training and Matricaria chamomilla flowers hydroethanolic extract on cognitive deficit in type 2 diabetes rats. Biomed Pharmacotherap Biomed Pharmacotherapie.

[19] Fazelzadeh M, Afzalpour ME, Fallah Mohammadi Z, Falah Mohammadi H (2021). The effects of voluntary complex and regular wheel running exercises on the levels of 8-oxoguanine DNA glycosylase, semaphorin 3B, H2O2, and apoptosis in the hippocampus of diabetic rats. Brain Behav.

[20] Hasanein P, Shahidi S (2012). Preventive effect of *Teucrium polium* on learning and memory deficits in diabetic rats. Med Sci Monit.

[21] Koocheki A, Tabrizi L, Mahallati MN (2007) The effects of irrigation intervals and manure on quantitative and qualitative characteristics of *Plantago ovata* and *Plantago psyllium*. Asian J Plant Sci

[22] Blumenthal M, Goldberg A, Brinckmann J (2000) Herbal medicine. Expanded commission E monographs. Integrative Medicine Communications

[23] Gibb RD, McRorie JW, Russell DA, Hasselblad V, D’Alessio DA (2015). Psyllium fiber improves glycemic control proportional to loss of glycemic control: a meta-analysis of data in euglycemic subjects, patients at risk of type 2 diabetes mellitus, and patients being treated for type 2 diabetes mellitus. Am J Clin Nutr.

[24] Zarvandi M, Rakhshandeh H, Abazari M, Shafiee-Nick R, Ghorbani A (2017). Safety and efficacy of a polyherbal formulation for the management of dyslipidemia and hyperglycemia in patients with advanced-stage of type-2 diabetes. Biomed Pharmacother.

[25] Lin J-Y, Kuo W-W, Baskaran R, Kuo C-H, Chen Y-A, Chen WS-T, Ho T-J, Day CH, Mahalakshmi B, Huang C-Y (2020). Swimming exercise stimulates IGF1/PI3K/Akt and AMPK/SIRT1/PGC1α survival signaling to suppress apoptosis and inflammation in aging hippocampus. Aging (Albany NY).

[26] Vaisy M, Szlufcik K, De Bock K, Eijnde BO, Van Proeyen K, Verbeke K, Van Veldhoven P, Hespel P (2011). Exercise-induced, but not creatine-induced, decrease in intramyocellular lipid content improves insulin sensitivity in rats. J Nutr Biochem.

[27] Luo C, Ke Y, Yuan Y, Zhao M, Wang F, Zhang Y, Bu S (2016). A novel herbal treatment reduces depressive-like behaviors and increases brain-derived neurotrophic factor levels in the brain of type 2 diabetic rats. Neuropsychiatr Dis Treat.

[28] Lalonde R, Lewis T, Strazielle C, Kim H, Fukuchi K (2003). Transgenic mice expressing the βAPP695SWE mutation: effects on exploratory activity, anxiety, and motor coordination. Brain Res.

[29] Hansen KF, Sakamoto K, Wayman GA, Impey S, Obrietan K (2010). Transgenic miR132 alters neuronal spine density and impairs novel object recognition memory. PLoS ONE.

[30] Ganji A, Salehi I, Nazari M, Taheri M, Komaki A (2017). Effects of *Hypericum scabrum* extract on learning and memory and oxidant/antioxidant status in rats fed a long-term high-fat diet. Metab Brain Dis.

[31] Ettcheto M, Sánchez-López E, Pons L, Busquets O, Olloquequi J, Beas-Zarate C, Pallas M, García ML, Auladell C, Folch J (2017). Dexibuprofen prevents neurodegeneration and cognitive decline in APPswe/PS1dE9 through multiple signaling pathways. Redox Biol.

[32] Lueptow LM (2017). Novel object recognition test for the investigation of learning and memory in mice. JoVE.

[33] Kassab S, Begley P, Church SJ, Rotariu SM, Chevalier-Riffard C, Dowsey AW, Phillips AM, Zeef LA, Grayson B, Neill JC (2019). Cognitive dysfunction in diabetic rats is prevented by pyridoxamine treatment. A multidisciplinary investigation. Mol Metab.

[34] Shekarian M, Komaki A, Shahidi S, Sarihi A, Salehi I, Raoufi S (2020). The protective and therapeutic effects of vinpocetine, a PDE1 inhibitor, on oxidative stress and learning and memory impairment induced by an intracerebroventricular (ICV) injection of amyloid beta (aβ) peptide. Behav Brain Res.

[35] Cavalcanti CL, Gonçalves MCR, Alves AF, de Araújo EV, Carvalho JLP, Lins PP, Alves RC, Soares NL, Pordeus LCM, Aquino JS (2020). Antidepressant, anxiolytic and neuroprotective activities of two zinc compounds in diabetic rats. Front Neurosci.

[36] Ahmadi N, Safari S, Mirazi N, Karimi SA, Komaki A (2021). Effects of vanillic acid on Aβ1-40-induced oxidative stress and learning and memory deficit in male rats. Brain Res Bull.

[37] Karimi SA, Salehi I, Taheri M, Faraji N, Komaki A (2020) Effects of regular exercise on diabetes-induced memory deficits and biochemical parameters in male rats. J Mol Neurosci. 1–8 10.1007/s12031-020-01724-3 33000398

[38] Shahidi S, Asl SS, Komaki A, Hashemi-Firouzi N (2018). The effect of chronic stimulation of serotonin receptor type 7 on recognition, passive avoidance memory, hippocampal long-term potentiation, and neuronal apoptosis in the amyloid β protein treated rat. Psychopharmacology.

[39] Ghaderi A, Karimi SA, Talaei F, Shahidi S, Faraji N, Komaki A (2020). The effects of aqueous extract of *Origanum vulgare* on learning and memory in male rats. J Herbmed Pharmacol.

[40] Shiri M, Komaki A, Oryan S, Taheri M, Komaki H, Etaee F (2017). Effects of cannabinoid and vanilloid receptor agonists and their interaction on learning and memory in rats. Can J Physiol Pharmacol.

[41] Liu K-F, Niu C-S, Tsai J-C, Yang C-L, Peng W-H, Niu H-S (2019) Comparison of area under the curve in various models of diabetic rats receiving chronic medication. Arch Med Sci 15 10.5114/aoms.2019.91471 PMC9266878 35832712

[42] Karimi SA, Salehi I, Taheri M, Faraji N, Komaki A (2021). Effects of regular exercise on diabetes-induced memory deficits and biochemical parameters in male rats. J Mol Neurosci.

[43] Sanjana F, Delgorio PL, Hiscox LV, DeConne TM, Hobson JC, Cohen ML, Johnson CL, Martens CR (2021). Blood lipid markers are associated with hippocampal viscoelastic properties and memory in humans. J Cereb Blood Flow Metab.

[44] Zhang P-A, Sun Q, Li Y-C, Weng R-X, Wu R, Zhang H-H, Xu G-Y (2020) Overexpression of purinergic P2X4 receptors in hippocampus rescues memory impairment in rats with type 2 diabetes. Neurosci Bull 1–14 10.1007/s12264-020-00478-7 PMC7340685 32198702

[45] Dun C, Liu J, Qiu F, Wu X, Wang Y, Zhao Y, Gu P (2016). Effects of *Astragalus polysaccharides* on memory impairment in a diabetic rat model. Neuropsychiatr Dis Treat.

[46] Pugazhenthi S, Qin L, Reddy PH (2017). Common neurodegenerative pathways in obesity, diabetes, and Alzheimer's disease. Biochimica et biophysica acta (BBA) Mol Basis Dis.

[47] Huang C-C, Chung C-M, Leu H-B, Lin L-Y, Chiu C-C, Hsu C-Y, Chiang C-H, Huang P-H, Chen T-J, Lin S-J (2014). Diabetes mellitus and the risk of Alzheimer’s disease: a nationwide population-based study. PLoS ONE.

[48] Weinstein G, Maillard P, Himali JJ, Beiser AS, Au R, Wolf PA, Seshadri S, DeCarli C (2015). Glucose indices are associated with cognitive and structural brain measures in young adults. Neurology.

[49] Verdile G, Keane KN, Cruzat VF, Medic S, Sabale M, Rowles J, Wijesekara N, Martins RN, Fraser PE, Newsholme P (2015). Inflammation and oxidative stress: the molecular connectivity between insulin resistance, obesity, and Alzheimer’s disease. Mediators Inflamm.

[50] Kumari M, Brunner E, Fuhrer R (2000). Minireview: mechanisms by which the metabolic syndrome and diabetes impair memory. J Gerontol A Biol Sci Med Sci.

[51] Nitta A, Murai R, Suzuki N, Ito H, Nomoto H, Katoh G, Furukawa Y, Furukawa S (2002). Diabetic neuropathies in brain are induced by deficiency of BDNF. Neurotoxicol Teratol.

[52] Gasecka A, Siwik D, Gajewska M, Jaguszewski MJ, Mazurek T, Filipiak KJ, Postuła M, Eyileten C (2020). Early biomarkers of neurodegenerative and neurovascular disorders in diabetes. J Clin Med.

[53] Amaral LSdB, Souza CS, Volpini RA, Shimizu MHM, de Bragança AC, Canale D, Seguro AC, Coimbra TM, de Magalhães ACM, Soares TdJ (2018). Previous exercise training reduces markers of renal oxidative stress and inflammation in streptozotocin-induced diabetic female rats. J Diabetes Res.

[54] Mehta BK, Singh KK, Banerjee S (2019). Effect of exercise on type 2 diabetes-associated cognitive impairment in rats. Int J Neurosci.

[55] Gomez-Pinilla F, Hillman C (2013). The influence of exercise on cognitive abilities. Compr Physiol.

[56] Tang L, Kang Y-T, Yin B, Sun L-J, Fan X-S (2017). Effects of weight-bearing ladder and aerobic treadmill exercise on learning and memory ability of diabetic rats and its mechanism. Zhongguo ying yong sheng li xue za zhi Zhongguo yingyong shenglixue zazh Chin J Appl Physiol.

[57] Chieffi S, Messina G, Villano I, Messina A, Esposito M, Monda V, Valenzano A, Moscatelli F, Esposito T, Carotenuto M (2017). Exercise influence on hippocampal function: possible involvement of orexin-A. Front Physiol.

[58] de Senna PN, Xavier LL, Bagatini PB, Saur L, Galland F, Zanotto C, Bernardi C, Nardin P, Gonçalves CA, Achaval M (2015). Physical training improves non-spatial memory, locomotor skills and the blood brain barrier in diabetic rats. Brain Res.

[59] Rodríguez-Morán M, Guerrero-Romero F, Lazcano-Burciaga G, (1998). Lipid-and glucose-lowering efficacy of *Plantago Psyllium* in type II diabetes. J Diabetes Complications.

[60] Ziai SA, Larijani B, Akhoondzadeh S, Fakhrzadeh H, Dastpak A, Bandarian F, Rezai A, Badi HN, Emami T (2005). Psyllium decreased serum glucose and glycosylated hemoglobin significantly in diabetic outpatients. J Ethnopharmacol.

