# Correction to: Swimming training and *Plantago psyllium* ameliorate cognitive impairment and glucose tolerance in streptozotocin–nicotinamide-induced type 2 diabetic rats

**DOI:** 10.1186/s12576-022-00838-0

**Published:** 2022-08-08

**Authors:** Hesam Parsa, Zahra Moradi-Khaligh, Sara Rajabi, Kamal Ranjbar, Alireza Komaki

**Affiliations:** 1 Department of Exercise Physiology, Faculty of Sport Sciences, Bu-Ali Sina University, Hamedan, Iran; 2 Department of Physical Education and Sport Science, Islamic Azad University, Bandar Abbas Branch, Bandar Abbas, Iran; 3 Neurophysiology Research Center, Hamadan University of Medical Sciences, Hamadan, Iran

## Correction to: The Journal of Physiological Sciences (2021) 71:37 https://doi.org/10.1186/s12576-021-00823-z

Following publication of the original article [1], the co-author “Kamal Ranjbar” would like to remove the following affiliation “Neurophysiology Research Center, Hamadan University of Medical Sciences, Hamadan, Iran”.

The original article has been corrected.
